# A translational approach to understanding anxiety: the limitations, strengths of differences. A commentary on Drzewiecki & Fox (2024)

**DOI:** 10.3758/s13415-024-01182-z

**Published:** 2024-03-19

**Authors:** Felippe Espinelli Amorim, Morgane Colom, Amy L. Milton

**Affiliations:** Department of Psychology, University of Cambridge, Downing Site, Cambridge, CB2 3EB UK

**Keywords:** Animal models, Anxiety, Emotion

Anxiety disorders affect millions of people worldwide, with the World Health Organization estimating that 301 million people globally have lived experience of anxiety disorders. However, despite their prevalence and impact on quality of life, currently available treatments are effective for only typically 50% of patients. Furthermore, although we know that the patient population is heterogeneous, current treatment strategies do not uniformly take this into account. Ideally, patients would have access to a wide range of therapies, and clinicians would be able to identify the right treatment for each patient, every time. These sentiments are shared by Drzewiecki & Fox (2024), who put forward the case that cross-species translational models, driven by an understanding of the processes that underlie anxiety disorders, will be critical for progress in this regard. We agree that cross-species, fully translational models are of great value to understanding the processes that underlie mental health disorders. Although there are differences between species (and the approaches taken by preclinical and clinical researchers), these differences can be not only potential limitations, but also strengths.

In their article, Drzewiecki and Fox (2024) address a major issue in the field, which is that the definition of terms, such as “fear,” “threat,” and “anxiety,” are used differently in the preclinical and clinical literatures, and sometimes differently between research groups within the same field. A common language would be immensely helpful in facilitating interaction between basic scientists and clinician researchers (Milton & Holmes, 2018). Drzewiecki & Fox sidestep the problem of multiple definitions by instead focusing on *processes* and affective states, rather than disorders. This allows them to embrace the heterogeneity of presentation of symptoms in anxiety disorders and relate this to heterogeneity in neural mechanisms. Thus, rather than individual differences being problematic, considered as “noise” to an otherwise consistent diagnosis, this more Research Domain Criteria (RDoC) influenced approach takes the potential limitation of heterogeneity and puts it to good explanatory use. These differences might be most fruitfully examined with the use of theory-driven computational modelling. Drzewiecki & Fox argue that this approach, which has considerably advanced our understanding of reinforcement learning, should be applied to the study of fear and anxiety, and particularly the heterogeneous neural computations driving the selection of anxiety-triggered responses across species and assays.

However, this focus on heterogeneity is not intended to underplay the fact that despite differences between species, the affective states relating to fear and anxiety are observed throughout the animal kingdom. All animals display specific and adaptive defensive actions that guide the individual towards survival. These processes are deeply rooted in our phylogenetic past and are remarkably conserved in all organisms across the phylogenetic tree, from single-celled protozoa to mammals. Evolutionary pressures have shaped the circuitry supporting fear responses, which is largely conserved amongst vertebrates. Theoretically, this suggests that observations from one species can be translated to another, and legitimises the use of animal models to understand the processes driving the choice of flexible defensive responses. As described by Drzewiecki & Fox, research in rodents gives invaluable insight into the distributed and competitive microcircuitry involved in selecting appropriate actions in an ever-changing and dynamic environment. However, the differences in processing in neural circuits contributing to the heterogeneity in subjective experiences of fear and anxiety also need to be validated in humans. The direct comparison of fear between human and rodent laboratory experiments is challenging because of the drastically different methods used to instantiate and measure defensive responses in both settings.

Drzewiecki & Fox make the persuasive case that designing more ecologically valid procedures, such as two-dimensional computerised environments featuring virtual predators, would allow researchers to overcome methodological divergences between humans and animals by diversifying the behavioural responses shown by humans. We agree and further argue that three-dimensional virtual reality procedures in which participants could navigate in a more immersive, complex environment and react to realistic stimuli would elicit an even broader action repertoire and facilitate the translation with rodent work. In most studies utilising virtual tasks, brain activity is recorded whilst the participant is stationary; however, the recent development of wearable magnetoencephalography devices (Topalovic et al., 2023) has the potential to open up remarkable avenues for acquiring brain activity in freely moving humans.

Drzewiecki & Fox further assert that sometimes differences can be an advantage. For a phenomenon as complex and heterogeneous as anxiety, no single behavioural assay can fully describe it. For example, to overcome the lack of expression of subjective feelings in animal models, behavioural outcomes, such as freezing and darting, are used as measures related to fear and anxiety. These do not capture the full range of defensive responses but do offer advantages in terms of the molecular and circuit-level neurobiological techniques that can be used in animal models. Thus, the authors argue for the necessity of a multilevel approach to improve this translational aspect of neuroscience, ranging from animal models to examine fear and anxiety neurobiology to computational models to connect studies across species (Neville et al., 2023). They also advocate for the use of specialised models that share specific characteristics with humans to overcome some limitations of standard nonhuman animal models. For example, they suggest that the spiny mouse (*Acomys cahirinus*), which unusually for rodents, experiences a menstrual cycle, can be used to understand the relationship between the menstrual cycle and anxiety. While we agree that using animal models with different specific characteristics can improve our understanding of anxiety, we do have some reservations with this approach. A major drawback is the risk of reducing generalisability and another that it is difficult to measure the translational validity of a finding if it can only be described under certain assumptions and specific conditions of one animal species. However, considering that preclinical animal research has been criticised for its lack of external validity (Pound & Ritskes-Hoitinga, 2018), it may be that greater diversity in animal models would be an advantage, as long as it is made clear exactly what each model does, and does not, model (Milton & Holmes, 2018).

Ultimately, as asserted by the statistician George Box, “all models are wrong.” A key point made by Drzewiecki & Fox, which we agree with, is that the choice of model needs to fit the question. Iterative development of translational and reverse translational models, facilitated by greater dialogue and collaboration between basic scientists and clinician researchers, will help us to identify the core common mechanisms that underlie fear and anxiety and those that contribute to the heterogeneity of presentation in humans (and animals). Ultimately, understanding phenomena as complex as mental health disorders, including the anxiety disorders that are the focus of this review, requires a multilevel approach that will allow an understanding of mechanisms operating at the cellular, circuit, systems, and behavioural levels. Different animal models have a key role to play in this endeavour, and being clear and honest about their strengths and weaknesses will help to ensure that, despite all models being wrong, that in the right circumstances, they are useful.
